# Radical surgery versus organ preservation via short-course radiotherapy followed by transanal endoscopic microsurgery for early-stage rectal cancer (TREC): a randomised, open-label feasibility study

**DOI:** 10.1016/S2468-1253(20)30333-2

**Published:** 2020-12-11

**Authors:** Simon P Bach, Alexandra Gilbert, Kristian Brock, Stephan Korsgen, Ian Geh, James Hill, Talvinder Gill, Paul Hainsworth, Matthew G Tutton, Jim Khan, Jonathan Robinson, Mark Steward, Christopher Cunningham, Bruce Levy, Alan Beveridge, Kelly Handley, Manjinder Kaur, Natalie Marchevsky, Laura Magill, Ann Russell, Philip Quirke, Nicholas P West, David Sebag-Montefiore, TREC collaborators, Gina Brown, Peter Antonio, Alex Vince, Nick Hilken, Chakanaka Sidile, Adrian Wilcockson, Richard Peto, Tom Crosby, Brendan Moran, Julie Olliff, Katti Ashok, Simone Slawik, Andrew Smethurst, Rajaram Sripadam, Veena Tagore, Monica Terlizzo, Bearn Philip, Robert Davies, Susan Dodd, Sharadah Essapen, Pasha Nisar, Alexandra Stewart, Jonathan Trickett, Bansal Ashish, Peter Billings, Palanichamy Chandran, Conor Corr, Edward Favill, Simon Gollins, Peter Marsh, Andrew Maw, Rakha Neupane, Ramesh Rajagopal, Rachel Cooper, John Griffith, Paul Hatfield, Andy Lowe, Julian Ostrowski, Jonathan Robinson, Rhian Simpson, Richard Adams, Robert Bleehen, Michael Davies, Meleri Morgan, Darren Boone, Nicola Lacey, Ian Seddon, Bruce Sizer, Helen Stunell, Shaobin Wu, Maher Hadaki, Dominic Blunt, Susan Cleator, Ara Darzi, Robert Goldin, Paul Ziprin, Mike Dobson, Mark Pitt, Shabbir Susnerwala, Deborah Williamson, Georgina Howarth, Stephen Lee, Paul Wright, Tim Hoare, Alan Horgan, Fiona McDonald, Stephanie Needham, John Scott, Timothy Simmons, Debashis Biswas, James Hernon, Gaurav Kapur, Sandeep Kapur, James Sington, Christopher Speakman, William Stebbings, Stuart Williams, Madhavi Adusumalli, Anil Agarwal, David Borowski, Dharmendra Garg, Talvinder Gill, Mohammed Hegab, Catherine Hobday, Veena Rao, Jyotsna Shrimankar, Mohamed Tabaqchali, David Wilson, Oliver Jones, Neil Mortensen, Andrew Slater, Aron Szuts, Lai Wang, Bryan Warren, Andrew Weaver, Mukhtar Ahmad, Julian Alexander, Maxine Flubacher, David Tarver, Suhail Baluch, Richard Beable, David Cowlishaw, Antony Higginson, Prokopios Vogiatzis, Neil Cruickshank, Howard Joy, David Peake, Ulises Zanetto, Mark Saunders, Arthur Sun-Myint, Rajaram Sripadam, Rachel Cooper, Paul Hatfield, Mark Teo, Arthur Allan, Ian Geh, John Glaholm, Mark Goldstein, Rahul Hejmadi, Gerald Langman, Dion Morton, Cyril Nelson, Deborah Tattersall, Stephen Falk, Robert Longman, Huw Roach, Jamshed Shabbir, Golda Shelley-Fraser, Michael Thomas, Neil Cripps, Yasser Haba, Guy Harris, Max Hookway, Jay Simson, Angela Skull, Tijani Umar

**Affiliations:** a Cancer Research UK Clinical Trials Unit, University of Birmingham, Birmingham, UK; b Birmingham Clinical Trials Unit, University of Birmingham, Birmingham, UK; c Department of Colorectal Surgery, University Hospitals Birmingham NHS Foundation Trust, Birmingham, UK; d Department of Radiation Oncology, University Hospitals Birmingham NHS Foundation Trust, Birmingham, UK; e Leeds Institute of Medical Research, University of Leeds, Leeds Cancer Centre, Leeds, UK; f Department of Colorectal Surgery, Central Manchester University Hospitals NHS Foundation Trust, Manchester, UK; g Department of Colorectal Surgery, North Tees and Hartlepool NHS Foundation Trust, University Hospital of North Tees, Stockton-on-Tees, UK; h Department of Colorectal Surgery, Newcastle upon Tyne Hospitals NHS Foundation Trust, Newcastle upon Tyne, UK; i Department of Colorectal Surgery, East Suffolk and North Essex NHS Foundation Trust, Colchester Hospital, Colchester, Essex, UK; j Department of Colorectal Surgery, Portsmouth Hospital NHS Trust, Portsmouth, UK; k Department of Colorectal Surgery, Bradford Teaching Hospitals NHS Foundation Trust, Bradford, UK; l Department of Colorectal Surgery, Oxford University Hospitals NHS Foundation Trust, Oxford, UK; m Department of Colorectal Surgery, Western Sussex Hospitals NHS Foundation Trust, West Sussex, UK; n Department of Colorectal Surgery, Lancashire Teaching Hospitals NHS Foundation Trust, Lancashire, UK; o Patient representative, National Cancer Research Institute, London, UK; p Division of Pathology and Data Analytics, School of Medicine, Leeds University, Leeds, UK

## Abstract

**Background:**

Radical surgery via total mesorectal excision might not be the optimal first-line treatment for early-stage rectal cancer. An organ-preserving strategy with selective total mesorectal excision could reduce the adverse effects of treatment without substantially compromising oncological outcomes. We investigated the feasibility of recruiting patients to a randomised trial comparing an organ-preserving strategy with total mesorectal excision.

**Methods:**

TREC was a randomised, open-label feasibility study done at 21 tertiary referral centres in the UK. Eligible participants were aged 18 years or older with rectal adenocarcinoma, staged T2 or lower, with a maximum diameter of 30 mm or less; patients with lymph node involvement or metastases were excluded. Patients were randomly allocated (1:1) by use of a computer-based randomisation service to undergo organ preservation with short-course radiotherapy followed by transanal endoscopic microsurgery after 8–10 weeks, or total mesorectal excision. Where the transanal endoscopic microsurgery specimen showed histopathological features associated with an increased risk of local recurrence, patients were considered for planned early conversion to total mesorectal excision. A non-randomised prospective registry captured patients for whom randomisation was considered inappropriate, because of a strong clinical indication for one treatment group. The primary endpoint was cumulative randomisation at 12, 18, and 24 months. Secondary outcomes evaluated safety, efficacy, and health-related quality of life assessed with the European Organisation for Research and Treatment of Cancer (EORTC) QLQ C30 and CR29 in the intention-to-treat population. This trial is registered with the ISRCTN Registry, ISRCTN14422743.

**Findings:**

Between Feb 22, 2012, and Dec 19, 2014, 55 patients were randomly assigned at 15 sites; 27 to organ preservation and 28 to radical surgery. Cumulatively, 18 patients had been randomly assigned at 12 months, 31 at 18 months, and 39 at 24 months. No patients died within 30 days of initial treatment, but one patient randomly assigned to organ preservation died within 6 months following conversion to total mesorectal excision with anastomotic leakage. Eight (30%) of 27 patients randomly assigned to organ preservation were converted to total mesorectal excision. Serious adverse events were reported in four (15%) of 27 patients randomly assigned to organ preservation versus 11 (39%) of 28 randomly assigned to total mesorectal excision (p=0·04, χ^2^ test). Serious adverse events associated with organ preservation were most commonly due to rectal bleeding or pain following transanal endoscopic microsurgery (reported in three cases). Radical total mesorectal excision was associated with medical and surgical complications including anastomotic leakage (two patients), kidney injury (two patients), cardiac arrest (one patient), and pneumonia (two patients). Histopathological features that would be considered to be associated with increased risk of tumour recurrence if observed after transanal endoscopic microsurgery alone were present in 16 (59%) of 27 patients randomly assigned to organ preservation, versus 24 (86%) of 28 randomly assigned to total mesorectal excision (p=0·03, χ^2^ test). Eight (30%) of 27 patients assigned to organ preservation achieved a complete response to radiotherapy. Patients who were randomly assigned to organ preservation showed improvements in patient-reported bowel toxicities and quality of life and function scores in multiple items compared to those who were randomly assigned to total mesorectal excision, which were sustained over 36 months’ follow-up. The non-randomised registry comprised 61 patients who underwent organ preservation and seven who underwent radical surgery. Non-randomised patients who underwent organ preservation were older than randomised patients and more likely to have life-limiting comorbidities. Serious adverse events occurred in ten (16%) of 61 non-randomised patients who underwent organ preservation versus one (14%) of seven who underwent total mesorectal excision. 24 (39%) of 61 non-randomised patients who underwent organ preservation had high-risk histopathological features, while 25 (41%) of 61 achieved a complete response. Overall, organ preservation was achieved in 19 (70%) of 27 randomised patients and 56 (92%) of 61 non-randomised patients.

**Interpretation:**

Short-course radiotherapy followed by transanal endoscopic microsurgery achieves high levels of organ preservation, with relatively low morbidity and indications of improved quality of life. These data support the use of organ preservation for patients considered unsuitable for primary total mesorectal excision due to the short-term risks associated with this surgery, and support further evaluation of short-course radiotherapy to achieve organ preservation in patients considered fit for total mesorectal excision. Larger randomised studies, such as the ongoing STAR-TREC study, are needed to more precisely determine oncological outcomes following different organ preservation treatment schedules.

**Funding:**

Cancer Research UK.

## Introduction

Radical surgical resection adhering to the principles of total mesorectal excision is the standard of care for localised rectal cancer, with selective use of pre-operative chemoradiotherapy reserved for patients with locally advanced disease.^1^ Although total mesorectal excision provides effective local tumour control, patients must accept a 2% mortality risk and the prospect of considerable short-term morbidity.^2^ Cancer survivors report long-term bowel, bladder, and sexual dysfunction following total mesorectal excision that impairs quality of life (QOL).^3^, ^4^

**Research in context**

**Evidence before this study**

Radical surgery to remove the rectum, adhering to the principles of total mesorectal excision, without pre-operative radiotherapy, is the standard of care for early-stage rectal cancer. However, radical surgery has measurable mortality, substantial morbidity, and a considerable impact on patients’ quality of life (QOL). Patient groups and health-care professionals both recognise the potential benefits of an organ preservation approach for the treatment of early-stage rectal cancer to reduce the morbidity associated with radical surgery without compromising oncological outcomes, but there is currently a lack of high-quality, prospective, randomised evidence to justify adoption of this approach. Organ preservation strategies commonly use a combination of radiotherapy and local excision. The feasibility of primary organ preservation with chemoradiotherapy is not in doubt, but the cumulative toxicity arising from multiple treatments is problematic, particularly for patients who do not achieve organ preservation, and adversely affects patients’ health-related QOL (HRQOL). Furthermore, to the best of our knowledge, no randomised study has compared an organ preservation strategy with total mesorectal excision alone. The GRECCAR 2 trial enrolled patients with small T2 and T3 rectal cancer following a good clinical response to chemoradiotherapy, randomly assigning patients to undergo either radical surgery or transanal local excision (transanal endoscopic microsurgery). Conversion to radical surgery was advised for patients with ypT2 or more advanced stage cancer. A composite endpoint of death, cancer recurrence, and treatment-related morbidity at 2 years concluded that organ preservation was not superior to chemoradiotherapy plus radical surgery due to the cumulative toxicities of multiple treatments. Further exploratory analysis by treatment received suggested lower rates of faecal incontinence in patients who achieved organ preservation at 2 years. Notably, few studies have reported longer-term toxicity rates or patient-reported outcomes including HRQOL following organ preservation, particularly in a randomised setting against total mesorectal excision surgery alone. This limits our ability to extrapolate available data on HRQOL to guide patient choices.

**Added value of this study**

The TREC study demonstrates the feasibility of randomly assigning patients with early-stage rectal cancer to a multimodality organ preservation strategy (incorporating short-course radiotherapy and transanal endoscopic microsurgery) versus radical surgery without radiotherapy. The comparison of organ preservation with radical surgery showed some benefits of organ preservation with respect to fewer serious surgical complications, low acute patient-reported toxicity, and little impact on QOL and function at 3 months. Sustained benefits for up to 3 years in overall QOL, social function, body image, and decreased embarrassment about bowel function were also observed with organ preservation. The risk of unsalvageable local recurrence was low in TREC.

**Implications of all the available evidence**

The findings of the TREC study suggest that short-course radiotherapy and transanal endoscopic microsurgery is associated with lower acute and late patient-reported side-effects than total mesorectal excision (the standard of care), and consequently has a minimal impact on patients’ QOL. The high levels of compliance, low toxicity, significant downstaging, and high rates of organ preservation achieved in the TREC study support further evaluation of short-course radiotherapy to achieve primary organ preservation in patients with early-stage rectal cancer alongside other measures that could reduce the adverse effects of treatment, such as smaller risk-adapted radiotherapy fields, non-operative management of complete response, and a biomarker-driven approach to initiating surgical intervention. Such measures should be implemented in the context of well conducted clinical research, such as the ongoing STAR-TREC study, as larger randomised trials are required to establish the oncological safety of organ preservation.

25% of total mesorectal excision operations done in the UK are for non-irradiated T1 and T2 rectal tumours.^2^ Interest in primary organ preservation for early-stage rectal cancer is gathering momentum but there is a lack of high quality, prospective, randomised evidence to justify its adoption.^5^, ^6^ Primary organ preservation in early-stage rectal cancer aims to preserve the rectum and its function. This approach can involve radiotherapy, local excision, or a combination of treatment approaches. Local transanal surgical excision of early-stage rectal cancer can achieve organ preservation, but overall oncological outcomes are compromised.^1^, ^7^, ^8^ The risk of local recurrence following transanal excision is associated with the presence of specific high-risk histopathological features that are only evaluable, and thereby appreciated, once the tumour is removed.^8^ Chemoradiotherapy followed by transanal local excision is an experimental approach to improve outcomes for primary organ preservation in early-stage rectal tumours, but multiple treatments can give rise to cumulative toxicities that detract from the benefits of organ preservation.^9^, ^10^, ^11^ The efficacy of pre-operative short-course radiotherapy compared with chemoradiotherapy for prevention of local relapse is supported by two phase 3 trials in patients with radically excised rectal cancer, where short-course radiotherapy showed benefits in terms of reduced toxicity.^12^, ^13^ Short-course radiotherapy with delayed local excision provided safe and effective primary organ preservation in a cohort of frail, elderly patients with T1 and T2 N0 rectal cancer,^14^ but excessive surgical morbidity was reported in another study.^15^

To our knowledge, TREC is the first study to randomly assign patients with early-stage rectal cancer to organ preservation (via short-course radiotherapy and delayed transanal endoscopic microsurgery) or radical surgery (total mesorectal excision) without preoperative chemoradiotherapy. TREC was designed to assess the feasibility of recruiting patients to a larger randomised phase 3 trial comparing these different treatment approaches.^16^ This study was designed to address the lack of randomised evidence describing the trajectory of symptomatic toxicity and health-related quality of life (HRQOL) following organ preservation and total mesorectal excision. The aim of the TREC study was to determine the feasibility of randomisation between two markedly different treatment options. We also aimed to explore the impact of the novel organ preservation intervention to inform the design of a larger phase 3 study comparing radical surgery with organ preservation.

## Methods

### Study design and participants

TREC was a randomised, open-label, feasibility study done in the UK, at 21 tertiary referral centres specialising in the treatment of early-stage rectal cancer. TREC assessed the feasibility of randomly assigning eligible patients with early-stage rectal cancer to either total mesorectal excision, without pre-operative radiotherapy in accordance with NICE guidance,^6^ or a novel organ preservation strategy. A non-randomised registry was included to capture treatment outcomes in patients for whom randomisation was considered inappropriate, due to a strong clinical indication for one of the treatment options (eg, individuals considered unsuitable for total mesorectal excision due to frailty, comorbidity, or older age).

Eligible patients were aged 18 years or older with rectal adenocarcinoma staged T2 or lower, no lymph node involvement (ie, N0), with a maximum diameter of 30 mm or less, and no metastasis. Patients with a previous history of pelvic radiotherapy were excluded. Participants provided written informed consent and the study was done in accordance with the Declaration of Helsinki and Good Clinical Practice Guidelines. The trial was approved by the West Midlands, Black Country Research Ethics Committee (10/H1202/81).

### Randomisation and masking

All patients were reviewed by a colorectal cancer multidisciplinary team at each centre, and eligible patients who were considered equally suitable for total mesorectal excision or organ preservation were invited to enrol in the study and randomly assigned in a 1:1 ratio. Treatment allocation was done with a computer-based randomisation service accessed by telephone or web link. Patients and investigators were not masked to treatment allocation. Where the multidisciplinary team strongly preferred one of the two treatment groups the patients were invited to participate in the study via a non-randomised registry. Entry to the registry was by online or telephone registration before any treatment was initiated. At the outset of the study, eligible patients who declined randomisation were excluded from the study and offered standard treatment, but a protocol amendment on May 8, 2013, allowed patients who were particularly reluctant to undergo radical surgery or formation of a stoma to choose organ preservation through the non-randomised registry.

### Procedures

Baseline investigations included colonoscopy, tumour biopsy, high-resolution pelvic MRI, endorectal ultrasound (optional) and CT scan of the thorax, abdomen, and pelvis. Organ preservation consisted of three-dimensional pelvic conformal short-course radiotherapy followed by local excision. 25 Gy in five fractions was given over a period of 5–7 days with photon energies of at least 6 MV and a three or four field technique. The clinical radiotherapy target volume included the tumour, mesorectum, and internal iliac, obturator and presacral nodes, up to the level of S2/3 junction. Radiotherapy quality assurance was coordinated by the UK National Cancer Research Institute Radiotherapy Trials Quality Assurance team.

After an interval of 8–10 weeks following completion of short-course radiotherapy the area of the rectal wall affected by cancer was removed, with a 10 mm margin of normal mucosa, via transanal endoscopic microsurgery. The surgeon performed full thickness local disc excision of the tumour site, incorporating the muscularis propria with a small amount of mesorectal fat. Transanal endoscopic microsurgery specimens were pinned out, mucosal surface upwards, to identify the rim of surrounding normal tissue, before fixing intact. Standard radical surgery adhered to the principles of total mesorectal excision by low anterior resection or abdominoperineal excision where the primary tumour encroached upon the anal canal. Surgery comprised a minimally invasive, open, or hybrid approach. Specimens were classified according to TNM5, and pT1 cancers were substaged by the depth of the submucosal invasion. Central histopathological review was done for quality assurance and to score radiotherapy regression grade. Pathological complete response (ypT0) was designated by no visible tumour cells, despite embedding the entirety of the tumour and sectioning all blocks at three levels. Histopathological features considered high risk for local relapse following organ preservation were a maximum tumour diameter greater than 30 mm, cancer within 1 mm of the circumferential or deep resection margin, predominantly poor differentiation, presence of lymphatic or venous invasion (intramural or extramural), and tumour depth of invasion of submucosal tumour stage 3 (sm3) or greater.^8^ Patients with one or more of these high-risk features were considered for planned early conversion from organ preservation to total mesorectal excision surgery within 8 weeks.

Patients were followed up in the clinic at 3, 6, 12, 24, and 36 months; with carcinoembryonic antigen testing at 12, 24, and 36 months; and CT scan of the thorax, abdomen, and pelvis at 12 and 24 months. Additionally, patients undergoing organ preservation had white light endoscopy and MRI scans every 3 months for 2 years, then every 6 months for up to 3 years.

### Outcomes

The primary endpoint was cumulative recruitment of randomised patients, reported 12, 18, and 24 months from the date of first randomisation. Secondary endpoints comprised measures of safety, including 30-day and 6-month mortality, clinician-reported morbidity (Common Terminology Criteria for Adverse Events [CTCAE], version 4·0, reported 3 weeks after short-course radiotherapy and incidence of serious adverse events), patient-reported toxicity, and HRQOL assessed with validated European Organisation for Research and Treatment of Cancer (EORTC) QLQ C29 and C30 questionnaires measured at baseline, and at 3, 6, 12, 24, and 36 months in the intention-to-treat population.^17^, ^18^ Serious adverse events were defined as those that caused death, threat to life, prolonged hospital stay, readmission to hospital, or persistent disability. Secondary efficacy endpoints were tumour downstaging (including the incidence of histopathological features considered high risk for local recurrence following transanal excision alone), organ preservation rate, stoma rates, tumour recurrence, disease-free survival, overall survival, and HRQOL. Overall survival was calculated from the date of randomisation or registration to the date of death, and disease-free survival was calculated from the date of randomisation or registration to the date of death or disease recurrence, whichever occurred first. The site of recurrence at first relapse was grouped as isolated local (pelvic), local plus distant, or isolated distant, to determine recurrence-free survival.

### Statistical analysis

Statistical analysis was done at Birmingham Cancer Research UK Clinical Trials Unit and Birmingham Clinical Trials Unit with R software (version 3.5.2). TREC assessed the feasibility of developing multidisciplinary organ preservation teams, opening sites, and recruiting patients to a randomised trial design. The study was not formally powered for a cancer outcome and no a-priori recruitment target was set. The secondary endpoint of tumour downstaging was selected to show the effect size needed to produce a significant difference between randomised groups in the intention-to-treat population. As preoperative short-course radiotherapy reduces local recurrence after total mesorectal excision by 50%,^19^ we hypothesised that short-course radiotherapy with an interval of 8–10 weeks to transanal endoscopic microsurgery would reduce the incidence of high-risk histopathological features by 50% or more compared with non-irradiated, radical surgery. Histopathological features that were considered high risk for local recurrence were derived from previous studies in patients treated with transanal endoscopic microsurgery alone, as were estimates of prevalence.^8^ 46 randomised patients were required to show that short-course radiotherapy reduced the incidence of high-risk features from 75% to 35% (α=0·05, 80% power, one-tailed). Binary proportions were compared across randomised groups by χ^2^ test. Survival outcomes were estimated with the Kaplan-Meier method and randomised groups were compared with the log-rank test. Patients without the event in each outcome were censored at the last known event-free time. Outcomes for the non-randomised population are provided for hypothesis generation. Completion of patient-reported outcomes, and item and scale compliance were determined. EORTC-QLQ guidelines were used for analyses and management of missing data.^20^ All item responses were converted from a four-point Likert-type scale with linear transformation onto a 0–100 scale.^20^ Differences in mean scores were classified as a small change for 5–10 points, a moderate change for 10–20 points, or a large change for more than 20 points.^21^ Exploratory item-level probabilities of the reported benefit or detriment of an organ-sparing approach compared to radical surgery were calculated for each patient-reported outcome assessment point with a Bayesian model-based analysis, adjusting for assessment time, treatment allocation, and baseline patient-reported outcome score. Trial oversight was maintained by a combined trial steering committee and data monitoring committee.

This trial is registered with the ISRCTN Registry, ISRCTN14422743.

### Role of the funding source

Cancer Research UK reviewed, approved, and funded the study design (CRUK/09/032). The funder played no part in study design, data analysis, or writing of this report. SPB, KB, AG, NM, MK, LM, KH, DSM, PQ, and NPW accessed and verified the data. All authors had full access to all trial data and had final responsibility for the decision to submit for publication.

## Results

**Figure 1:**
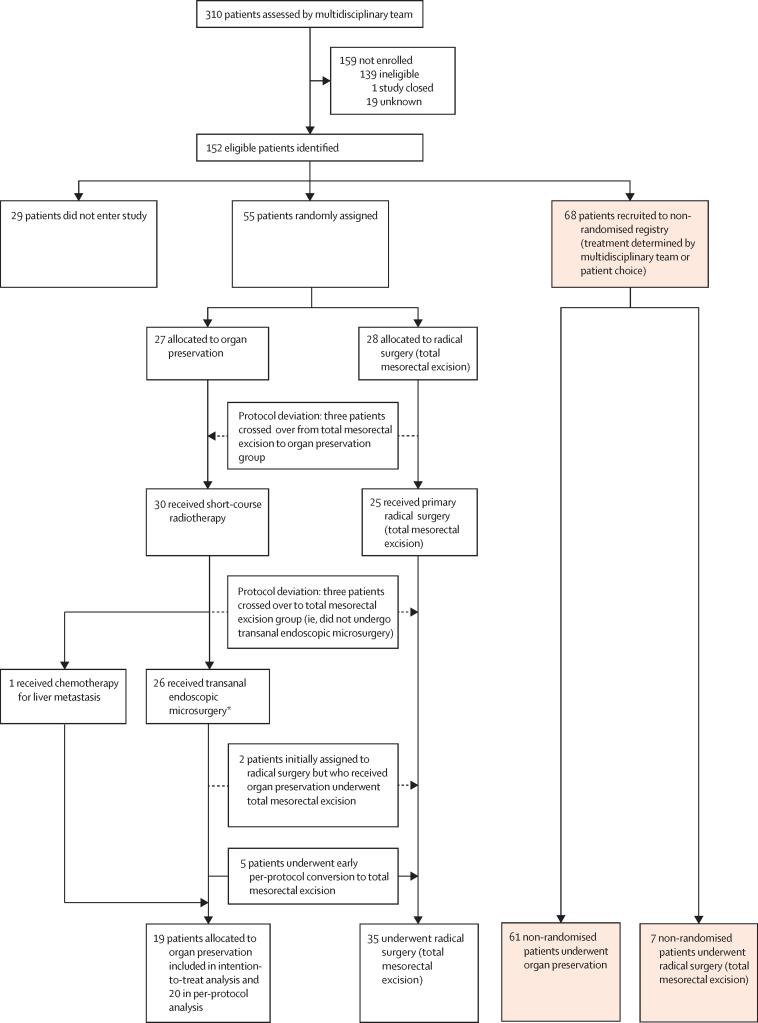
Trial profile *23 patients were randomly assigned to organ preservation while three crossed over from total mesorectal excision to organ preservation.

152 eligible patients with early-stage rectal cancer were identified at 21 UK sites; 14 recruiting sites opened during year 1, and seven sites joined during year 2. 123 (81%) patients entered the TREC study, whereas 29 declined (figure 1). Between Feb 22, 2012, and Dec 19, 2014, 55 patients consented to randomisation at 15 sites (appendix p 8); 27 patients were allocated to organ preservation and 28 to radical surgery (figure 1). Cumulative recruitment to the randomised study was as follows: 18 patients at 12 months, 31 at 18 months, 39 at 24 months, and 55 at 36 months. The date for final follow-up was April 19, 2018. Randomised patients were generally well matched in terms of age, sex, American Society of Anesthesiologists (ASA) physical status classification, and tumour stage (table 1). 68 patients were recruited to the non-randomised registry at 17 sites, with organ preservation preferred in 61 patients and radical total mesorectal excision surgery in seven (figure 1). Non-randomised patients who underwent organ preservation were generally older than randomised patients, more likely to have life-limiting comorbidities, and more often had T2 tumours (table 1). Six of 21 sites contributed non-randomised patients only. All sites that recruited more than three patients to TREC included patients for randomisation (appendix p 8).

**Table 1:** Baseline characteristics and treatment of intention-to-treat population

		**Randomised patients**		**Non-randomised patients**	
		Organ preservation (n=27)	Radical surgery (n=28)	Organ preservation (n=61)	Radical surgery (n=7)
Age, years		65 (52–79)	65 (49–83)	74 (53–89)	69 (53–73)
Sex					
	Male	19 (70%)	17 (61%)	39 (64%)	4 (57%)
	Female	8 (30%)	11 (39%)	22 (36%)	3 (43%)
ASA physical status classification					
	I	11 (41%)	13 (47%)	24 (39%)	4 (57%)
	II	14 (52%)	11 (39%)	23 (38%)	2 (29%)
	III	2 (7%)	4 (14%)	13 (21%)	0
	Missing	··	··	1 (2%)	1 (4%)
Distance from lower border of tumour to anal verge, mm		60 (40–80)	60 (50–70)	60 (40–70)	55 (35–75)
Tumour position					
	Anterior	7 (27%)	10 (35%)	8 (15%)	1 (14%)
	Lateral	14 (54%)	5 (18%)	20 (36%)	3 (43%)
	Posterior	5 (19%)	12 (43%)	23 42%)	3 (43%)
	Not known	0	1 (4%)	4 (7%)	0
MRI T stage					
	Tx	1 (4%)	2 (7%)	6 (10%)	0
	T1	10 (37%)	5 (18%)	10 (16%)	0
	T2	16 (59%)	21 (75%)	45 (74%)	7 (100%)
Ultrasound stage					
	Tx	0	0	1 (2%)	0
	T1	9 (33%)	10 (36%)	16 (26%)	1 (14%)
	T2	10 (37%)	9 (32%)	31 (51%)	4 (57%)
	Not done	8 (30%)	9 (32%)	13 (21%)	2 (29%)
Sphincter preservation considered feasible					
	Yes	23 (85%)	25 (89%)	··	··
	No	4 (15%)	3 (11%)	··	··
Compliance with treatment allocation		23 (85%)	25 (89%)	55 (90%)	7 (100%)
SCRT commenced		27 (100%)	3 (11%)	60 (98%)	··
SCRT completed		27 (100%)	3 (11%)	60 (98%)	··
Primary surgery					
	TEM	23 (85%)	3 (11%)	55 (92%)	0
	Low anterior resection	3 (11%)	23 (82%)	0	7 (100%)
	Abdominoperineal excision	0	2 (7%)	0	0
	No surgery*	1 (4%)	0	5 (8%)	0
	Temporary stoma	2 (7%)	20 (71%)	0	5 (71%)
	Stoma for complications	1 (4%)	3 (11%)	2	1 (14%)
Planned early conversion to TME following organ preservation		5 (19%)	2 (7%)	4 (7%)	··
Second surgery (planned early conversion following TEM)					
	Low anterior resection	3	0	1	··
	Abdominoperineal excision	1	2	3	··
	Hartmann's	1	0	0	··
	Temporary stoma	3	0	1	··
Overall stoma rate					
	Permanent	2 (7%)	4 (14%)	3 (5%)	0
	Temporary	6 (22%)	23 (82%)	3 (5%)	6 (86%)
Organ preservation rate		19 (70%)	1 (3%)	56 (92%)	0 (0%)

ASA=American Society of Anesthesiologists. SCRT=short-course radiotherapy. TEM=transanal endoscopic microsurgery. TME=total mesorectal excision.

* No TEM done following complete clinical response of the primary tumour.

Compliance with the organ preservation treatment strategy was high for both randomised (23 [85%] of 27 patients) and non-randomised groups (55 [90%] of 61; table 1). Short-course radiotherapy was well tolerated, with four (15%) of 27 patients having one or more grade 3 adverse event, of which diarrhoea was the most common (appendix p 1). Three patients who were randomly assigned to total mesorectal excision refused this treatment allocation and instead received the organ preservation treatment (protocol deviation); two of these three patients reverted back to total mesorectal excision following transanal endoscopic microsurgery due to the presence of histopathological features considered high risk for local recurrence (figure 1). Patients randomly assigned to undergo primary total mesorectal excision were most frequently reconstructed by low anterior resection (23 [82%] of 28), with a temporary diverting stoma (20 [71%] of 28; table 1). The rate of abdominoperineal excision with total mesorectal excision was 14% (four of 28 patients) in the intention-to-treat population. Serious adverse events were reported in four (15%) of 27 patients randomly assigned to organ preservation versus 11 (39%) of 28 randomly assigned to radical surgery (table 2; p=0·04, χ^2^ test). One patient allocated to organ preservation who had total mesorectal excision as the first surgery (no transanal endoscopic microsurgery) died following anastomotic leakage (ypT2N0). Serious adverse events were reported for ten (16%) of 61 non-randomised patients allocated to undergo organ preservation and one (14%) of seven allocated to undergo total mesorectal excision (table 2).

**Table 2:** Serious adverse events in the intention-to-treat population

		**Randomised patients**		**Non-randomised patients**	
		Organ preservation (n=27)	Radical surgery (n=28)	Organ preservation (n=61)	Radical surgery (n=7)
30-day mortality		0	0	0	0
6-month mortality		1*	0	0	0
All serious adverse events		5	15	15	1
Number of patients with a serious adverse event†		4 (15%)	11 (39%)	10 (16%)	1 (14%)
Cause of serious adverse events reported					
	Abdominal pain	0	0	1	0
	Acute kidney injury	0	2	0	0
	Anastomotic leak	1*	2	2	1
	Anastomotic stricture	0	1	0	0
	Cardiac arrest	0	1	0	0
	Cardiac failure	0	0	1	0
	Diarrhoea	1	1	2	0
	Deep venous thrombosis	0	1	0	0
	Fever	0	0	1	0
	Fistula	0	0	1	0
	Incisional hernia	0	1	0	0
	Pancreatitis	0	1	0	0
	Paralytic ileus	0	1	1	0
	Pneumonia	0	2	2	0
	Rectal bleed	2	1	2	0
	Rectal pain	1	0	0	0
	Stoma oedema	0	1	0	0
	Stroke	0	0	2	0

Serious adverse events are classified as complications leading to either death, threat to life, prolonged hospital stay, readmission to hospital, or persistent disability.

* Primary surgery was low anterior resection.

† Comparison of proportion of randomised patients with one or more serious adverse events (p=0·04, χ^2^ test).

Organ preservation was achieved in 19 (70%) of 27 randomised patients and 56 (92%) of 61 non-randomised patients (table 1). Three patients (staged ypT1sm2N0, ypT1sm3N0, and ypT2N0) randomly assigned to organ preservation underwent total mesorectal excision by low anterior resection following short-course radiotherapy without undergoing transanal endoscopic microsurgery (protocol deviations); in one, the transanal endoscopic microsurgery equipment could not reach the tumour, while in the other two the tumour was considered too large for transanal endoscopic microsurgery. Early per-protocol conversion from organ preservation to total mesorectal excision followed histopathological evaluation of the transanal endoscopic microsurgery specimen in five (19%) of 27 patients (table 1); there was no evidence of residual tumour in three patients, while two were staged ypT2N0.

Histopathological evaluation of randomised transanal endoscopic microsurgery specimens revealed that seven (26%) of 27 patients achieved a pathological complete response, and one achieved a clinical complete response (without transanal endoscopic microsurgery); therefore, eight (30%) of 27 patients randomly assigned to organ preservation achieved a complete response (table 3). The remaining patients were staged pT1 (six [22%] of 27), pT2 (eight [30%] of 27), and pT3 (five [19%] of 27). Of the 26 patients who underwent resection, resection margins were clear of cancer in 23 (88%) cases; three (12%) demonstrated adenocarcinoma up to 1 mm of the deep margin. Histopathological examination revealed that 14 (50%) of 28 patients randomly assigned to total mesorectal excision (ie, who did not undergo radiotherapy) were pT1, 13 (46%) of 28 were pT2, and one (4%) of 28 was pT3 (table 3). Microscopic lymph node metastasis was detected in four (14%) of 28 patients. Resection margins were clear of cancer (≥1 mm) in 26 (96%) of 27 patients who had total mesorectal excision; an abdominoperineal excision was R1 at the circumferential margin. Histopathological features that would be considered high risk for local tumour recurrence if present following transanal endoscopic microsurgery alone were present in 24 (86%) of 28 patients randomly assigned to radical surgery (ie, who did not undergo radiotherapy), versus 16 (59%) of 27 randomly assigned to organ preservation (p=0·03, χ^2^ test; table 3). 25 (41%) of 61 non-randomised patients who underwent organ preservation achieved a complete response, while high-risk histopathological features were present in 24 (39%) of 61 (table 3).

**Table 3:** Histopathological tumour characteristics

		**Randomised patients**		**Non-randomised patients**	
		Organ preservation (n=27)	Radical surgery (n=28)	Organ preservation (n=61)	Radical surgery(n=7)
TNM (y)pT stage					
	0	7 (26%)	0	20 (33%)	0
	1	6 (22%)	14 (50%)	18 (30%)	1 (14%)
	2	8 (30%)	13 (46%)	12 (20%)	6 (86%)
	3	5 (19%)	1 (4%)	4 (7%)	0
	x	0	0	1 (2%)	0
Clinical complete response with no surgery		1 (4%)	0	5 (8%)	0
All cases with a complete response		8 (30%)	0	25 (41%)	0
Kikuchi substaging of pT1 tumours					
	Submucosal tumour stage 1	2 (33%)	2 (14%)	7 (39%)	0
	Submucosal tumour stage 2	1 (17%)	3 (21%)	6 (33%)	0
	Submucosal tumour stage 3	3 (50%)	9 (64%)	5 (38%)	1 (100%)
TNM pN stage					
	0	3 (11%)	23 (82%)	0	7 (100%)
	1	0	3 (10%)	0	0
	2	0	1 (4%)	0	0
	x	24 (89%)	1 (4%)	60 (100%)	0
Surgical resection margin <1 mm					
	Clear	23 (85%)	26 (93%)	49 (82%)	7 (100%)
	Involved adenoma	0	1 (4%)	1 (2%)	0
	Involved carcinoma (deep)	3 (11%)	1 (4%)	1 (2%)	0
	Involved carcinoma (mucosal)	0	0	0	0
	No surgery	1 (4%)	0	5 (8%)	0
	Missing	0	0	4 (6%)	0
Positive lymphatic invasion		0	5 (18%)	1 (2%)	1 (14%)
Positive vascular invasion		2 (8%)	7 (25%)	4 (7%)	0
Tumour differentiation					
	Well or moderately differentiated	17 (63%)	26 (93%)	31 (51%)	7 (100%)
	Poorly differentiated	2 (7%)	2 (7%)	3 (5%)	0
	Pathological complete response	7 (26%)	0	19 (31%)	0
	Complete response*	1 (4%)	0	5 (8%)	0
Tumour diameter, mm		3 (0–17)	10 (0–30)	4 (0–45)	8 (2–30)
Radiotherapy response (Dworak 5-point system)					
	Complete	7 (26%)	0	19 (31%)	··
	Good	5 (19%)	2 (67%)	15 (25%)	··
	Moderate	6 (22%)	0	7 (11%)	··
	Mild	4 (15%)	0	4 (7%)	··
	No surgery	1 (4%)	0	5 (8%)	··
	Missing	3 (11%)	1 (33%)	5 (8%)	··
High-risk histopathological feature present†		16/27 (59%)	24/28 (86%)	24/61 (39%)	7/7 (100%)

* Including pathological and clinical complete response.

† Comparison of proportion of randomised patients with one or more histopathological feature considered high risk for relapse following local excision (maximum tumour diameter >30 mm, R1 resection, predominantly poor differentiation, presence of lymphatic or venous invasion and depth of invasion ≥submucosal tumour stage 3; p=0·03, χ^2^ test).

**Figure 2:**
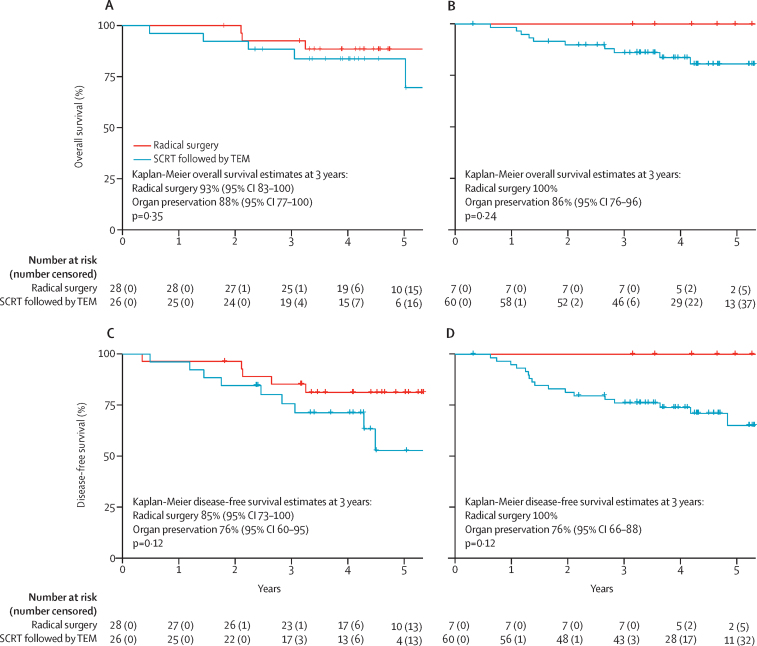
Overall survival and disease-free survival for randomised and non-randomised populations (A) Overall survival in the randomised population. (B) Overall survival in the non-randomised population. (C) Disease-free survival in the randomised population. (D) Disease-free survival in the non-randomised population. SCRT=short-course radiotherapy. TEM=transanal endoscopic microsurgery.

**Figure 3:**
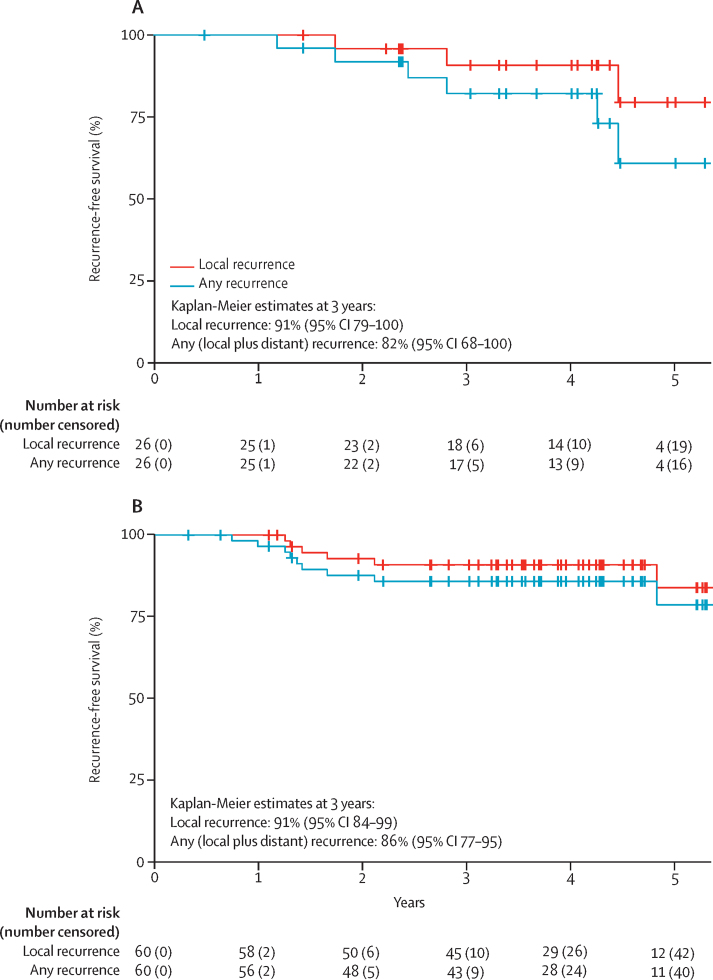
Cumulative risk of any local recurrence compared to risk of any recurrence (local or distant) with organ preservation therapy in intention-to-treat population (A) Randomised population. (B) Non-randomised population.

Median follow-up of randomised patients was 4·28 years (IQR 3·27–5·02). There was no significant difference between randomised groups with respect to either overall survival (hazard ratio [HR] 1·95 [95% CI 0·47–8·16]; p=0·35) or disease-free survival (2·32 [0·77–6·95]; p=0·12; figure 2). Local recurrence occurred in three (11%) of 27 patients randomly assigned to organ preservation and was isolated and technically salvageable in two (7%) of 27 patients; however, severe cardiovascular comorbidity prohibited surgical salvage in one individual (appendix p 2). All three patients who developed local recurrence had previously declined planned conversion to total mesorectal excision despite having high-risk transanal endoscopic microsurgery features: ypT3 in two patients and R1 in one patient (appendix p 2). No instances of local recurrence were recorded following primary total mesorectal excision. Isolated systemic metastasis developed in three (11%) of 27 patients allocated to organ preservation versus two (7%) of 28 allocated to total mesorectal excision (appendix p 2). Median follow-up of non-randomised patients was 4·07 years (IQR 3·19–4·69). Local recurrence occurred in six (10%) of 60 non-randomised patients allocated to organ preservation; recurrence was isolated in three patients (appendix p 3). Systemic recurrence developed in six (10%) of 60 non-randomised patients allocated to organ preservation. The proportion of patients free from local recurrence at 3 years following organ preservation was 91% (95% CI 79–100) in the randomised population and 91% (84–99) in the non-randomised population (figure 3).

**Figure 4:**
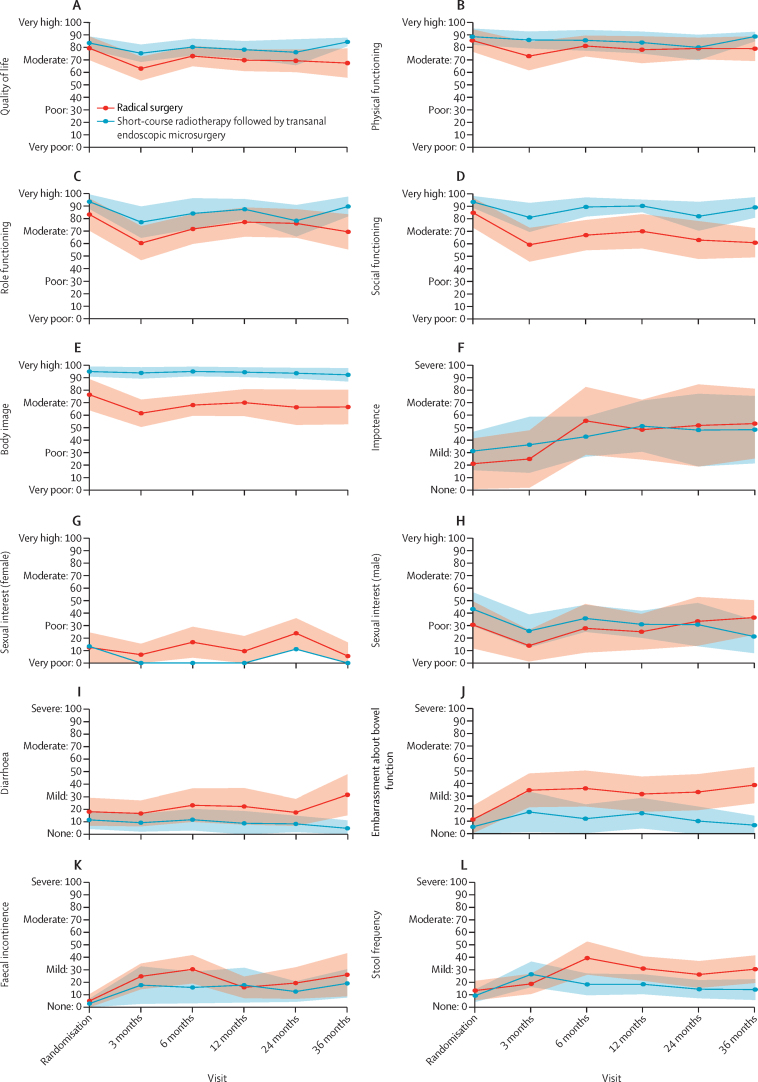
Change in EORTC QLQ C30 and CR29 over time for randomised patients Data are mean (95% CI). (A) Quality of life. (B) Physical functioning. (C) Role functioning. (D) Social functioning. (E) Body image. (F) Impotence. (G) Sexual interest (female). (H) Sexual interest (male). (I) Diarrhoea. (J) Embarrassment about bowel function. (K) Faecal incontinence. (L) Stool frequency. EORTC=European Organisation for Research and Treatment of Cancer.

Patient allocation and patient-reported outcome completion rates for randomised patients are provided in the appendix (p 9). Compliance with patient-reported outcomes was good (78·5% median completion rate; 64·0% 36-month response rate), with no difference in response rates or missing items between groups. Key patient-reported outcome data are presented for randomised patients in figure 4. A full summary of each patient-reported outcome item at each timepoint for randomised patients is provided in the appendix (pp 4–5), including a Bayesian model-based analysis (appendix p 6). Baseline questionnaires were completed following randomisation and patients had knowledge of their treatment allocation. Differences at baseline favoured organ preservation, for emotional (difference of 11·6 points) and role function (difference of 10·3 points), body image (difference of 18·5 points), and health anxiety (difference of 21·3 points).

During follow-up, patients randomly assigned to total mesorectal excision reported a deterioration in function scores at 3 months, with moderate differences between the two randomised groups favouring organ preservation for overall QOL, health anxiety, and physical, social, and role function (figure 4A–D). Patients randomly assigned to total mesorectal excision also demonstrated persistent moderate or large deteriorations in overall QOL, health anxiety, and role and social function, compared to baseline. The low body image scores noted preoperatively in the total mesorectal excision group at randomisation deteriorated further at 3 months and subsequently did not demonstrate recovery up to 36 months (figure 4E). Patients randomly assigned to organ preservation did not have any significant deterioration in pre-treatment QOL, health anxiety, and function (physical, role, social, emotional, and cognitive) over the 36-month study period. Minimal change in body image scores occurred in the organ preservation group and a large difference persisted compared with total mesorectal excision at 36 months (difference of 25·7 points).

For symptom items, there were no baseline differences in score (figure 4F, I–L). Compared with the organ preservation group, patients randomly assigned to total mesorectal excision showed a deterioration at 6 months in stool frequency, flatulence, incontinence, abdominal pain, and embarrassment about bowel function. Differences persisted at 36 months for overall stool frequency (difference of 16·3 points; figure 4L), stool frequency at night (difference of 25·4 points), and embarrassment about bowel function (difference of 31·7 points; figure 4J) in favour of organ preservation. There was also a moderate increase from baseline diarrhoea scores at 36 months for patients randomly assigned to total mesorectal excision (increase of 13·4 points) in comparison with those assigned to organ preservation (decrease of 6·8 points; figure 4I). Notably, the scores in the total mesorectal excision and organ preservation groups for faecal incontinence and flatulence were relatively similar at 36 months, with both groups reporting relatively mild symptoms (figure 4K). Urinary symptoms, including frequency and incontinence, remained stable during follow-up. Impotence scores deteriorated from baseline to 36 months (figure 4F) in both the randomised total mesorectal excision group (increase of 32·1 points) and the randomised organ preservation group (increase of 17·1 points). No marked changes occurred in female sexual items (figure 4G). The benefit of organ preservation in patient-reported outcome scores was supported by an exploratory Bayesian model-based analysis (appendix p 6). Higher probabilities towards benefit for organ preservation were observed with organ preservation in many items at baseline, as noted previously. Inferences were adjusted for baseline values. At 36 months there was a 90% or greater probability of superiority for organ preservation in many patient-reported outcome items, including overall QOL; role, social, and emotional function; body image; health anxiety; diarrhoea; stool frequency; embarrassment about bowel function; and urinary incontinence.

## Discussion

The TREC study demonstrates that it is feasible to randomly assign patients with early-stage rectal cancer to two markedly different treatment options: multimodality organ preservation treatment or total mesorectal excision with no preoperative radiotherapy. 81% of eligible patients approached for TREC took part and 36% were randomly assigned, highlighting the acceptability of an organ preservation approach if it is offered. All sites that included more than three patients were able to randomly assign patients, suggesting that clinical teams effectively adapted conventional clinical consultations to randomly assign patients to a study comparing two substantially different treatment approaches. The high proportion of non-randomised patients allocated to organ preservation indicates that referral patterns to specialist organ preservation practices were well established for frail, elderly, comorbid, and stoma-averse individuals with early-stage rectal cancer but underdeveloped in the wider patient population considered suitable for total mesorectal excision. Importantly, TREC was designed as a feasibility study, to introduce and evaluate a protocolised, multimodality organ preservation approach. The primary aim was to determine whether patients and clinicians would support randomisation between radical surgery and organ preservation, with secondary aims relating to safety, efficacy, and acceptability—key elements to inform the design of a future phase 3 trial.

Recent systematic reviews do not support adoption of an organ preservation approach for treatment of early-stage rectal cancer in fit patients because of insufficient high quality evidence.^22^, ^23^ In comparison with previous studies of chemoradiotherapy, the organ preservation approach followed in the TREC study has a number of strengths; we reported higher radiotherapy compliance (99% in randomised and non-randomised patients) than with chemoradiotherapy in previous studies (72–89%) and low acute toxicity (grade 3 adverse events: 15% *vs* 10–42%), alongside favourable organ preservation and complete response rates.^10^, ^11^, ^24^ The low acute toxicity rate is not only reflected by clinician-reported monitoring of serious adverse events, but importantly also by prospective assessment of patient-reported outcomes. As a feasibility study, TREC was not powered to undertake formal comparisons of specific long-term cancer outcomes, but the sample size allowed exploratory evaluation of downstaging of histopathological features that would be considered high risk for local recurrence if present following transanal endoscopic microsurgery alone.^8^ Introduction of short-course radiotherapy with an interval of 8–10 weeks to transanal endoscopic microsurgery was associated with significant tumour downstaging, corroborating an earlier report.^25^

To the best of our knowledge, we have, for the first time, shown consistent indications of benefit in multiple aspects of HRQOL and patient-reported toxicity with an organ preservation approach compared with total mesorectal excision alone, for up to 3 years. The risk of serious adverse events was lower with an organ preservation approach (15%) than with total mesorectal excision (39%), which is the current standard of care. The organ preservation rates observed with short-course radiotherapy are equivalent to those observed in other studies evaluating chemoradiotherapy and transanal endoscopic microsurgery.^10^, ^24^, ^26^ Results from non-randomised patients who underwent organ preservation, who were a decade older with increased comorbidities, were consistent with those obtained from the randomised patient population and also in accordance with our previous experience of using short-course radiotherapy and transanal endoscopic microsurgery to achieve organ preservation in elderly and frail patients.^14^ Several studies of organ preservation have recorded severe treatment-related toxicities that might be poorly tolerated in an older, frail patient population and so it was reassuring that in our study the tolerability and toxicity of short-course radiotherapy and transanal endoscopic microsurgery did not seem to be any worse for this group.^24^, ^27^ The high rates of organ preservation for non-randomised patients might reflect a clear reticence for total mesorectal excision to be either offered or accepted. It is likely that the threshold for converting patients to total mesorectal excision in the non-randomised organ preservation group was higher than in the randomised group as non-randomised patients were generally older, with comorbidities. Notably, this discrepancy in conversion rates did not lead to a marked increase in local recurrence. Future studies need to address the best approach to manage the decision to convert patients to total mesorectal excision.

Short-course radiotherapy and transanal endoscopic microsurgery, after an interval of 8–10 weeks, led to 30% of randomised patients and 41% of non-randomised patients achieving a complete response, which is similar to studies of chemoradiotherapy where 40–45% of patients achieved a complete response.^26^, ^27^ A 10-week interval from short-course radiotherapy to transanal endoscopic microsurgery was chosen to balance the potential benefits of waiting longer for optimal tumour regression with the risk of disease progression through treatment delay in a small proportion of patients who might be resistant to radiotherapy. At the inception of the TREC study the benefits of a good histopathological tumour response were appreciated,^28^ and the available evidence suggested that short-course radiotherapy produced downstaging after an interval to surgery of 10 days or more;^25^ however, the optimal timing for assessment of radiotherapy response was unknown. TREC used transanal endoscopic microsurgery routinely, as did other contemporary studies following reports of extremely poor correlation between clinical and histopathological staging in the context of a relatively short interval between radiotherapy and radical surgery.^29^ Emerging data on the oncological safety of non-operative approaches to rectal cancer treatment,^30^, ^31^ combined with the high complete response rates observed in this study and others,^10^, ^24^, ^26^ suggest that the adoption of a watch-and-wait strategy with selective use of transanal endoscopic microsurgery is feasible with the aim of further reducing treatment-related toxicity and improving HRQOL.^16^ However, the small sample size of TREC and the low number of events preclude meaningful analysis of oncological outcomes. We found no significant difference in either overall survival or disease-free survival between patients randomly assigned to organ preservation versus those randomly assigned to total mesorectal excision, in line with the GRECCAR 2 study,^11^ with very wide confidence intervals for the hazard ratios, reflecting the small sample size and relatively small number of events. A consistent proportion of patients relapse with distant metastasis regardless of primary therapy.^11^, ^32^ Isolated and technically salvageable local recurrence was detected in two (7%) of 27 patients randomly assigned to organ preservation, each associated with strong histological indicators for early conversion to total mesorectal excision (ypT3R0 and ypT2R1), but the patients had declined early conversion to total mesorectal excision and opted for close observation. Local recurrence might have been avoided with early conversion. These data emphasise the need for a phase 3 trial to more precisely define the risks and benefits associated with adopting an organ preservation approach.

Despite the small patient numbers, the patient-reported outcomes data reveal that an organ preservation approach is associated with consistent improvements in multiple symptom and function items 3 months after treatment, with longer-term benefits for overall QOL, social function, body image, and less embarrassment about bowel function. We found no evidence of acute deterioration in HRQOL or patient-reported toxicity with organ preservation versus total mesorectal excision.^10^, ^15^, ^24^ This exploratory analysis supports favourable longer-term patient-reported outcome scores seen at 1-year, 2-year, and 5-year follow-ups after organ preservation in various trials.^10^, ^33^ Our data provide an important contribution to the literature due to the absence of published randomised studies comparing patient-reported outcomes following total mesorectal excision and organ preservation. Although an unexpected finding, the lower baseline mean scores in a number of function items for the radical surgery group (completed after knowledge of randomisation status) might reflect our impression that the perceived risks of surgery in early-stage rectal cancer are higher than patients desire.

As a feasibility study, TREC provides the justification to further evaluate short-course radiotherapy and transanal endoscopic microsurgery versus radical surgery in a randomised setting. Although randomisation was feasible, our recruitment rate across centres indicated that recruitment to a future phase 3 trial should be international. Around the same time as the TREC study, a single-arm phase 2 trial done in the Netherlands (CARTS) had evaluated chemoradiotherapy followed by transanal endoscopic microsurgery.^9^ These studies provided the basis for the design of an international phase 2/3 trial (STAR-TREC) designed to randomly assign patients to radical surgery, organ preservation with short-course radiotherapy, and organ preservation with chemoradiotherapy.^16^ The external pilot (phase 2) demonstrated that it was possible to accelerate recruitment and the study progressed to phase 3 in 2020. The adoption of a more selective use of transanal endoscopic microsurgery, and a longer time interval to assess response in STAR-TREC were based on new data that became available after the completion of recruitment to TREC.

Our findings have some limitations: in a feasibility study the overall numbers of randomised patients are low and individual centres contributed relatively few patients. However, recruitment of a randomised patient population has considerable strengths and reduces many sources of bias. This dataset will help inform the choices of patients who would normally receive standard total mesorectal excision and are considering the option to undergo organ preservation instead. The non-randomised registry primarily set out to describe the tolerability of treatment and early outcomes for older individuals considered to be at high risk for total mesorectal excision as this population is generally under-represented in rectal cancer trials. Although eligibility for inclusion was identical to the randomised group, the results should not be directly extrapolated to patients considered fit for total mesorectal excision. It is nonetheless reassuring that consistent trends were demonstrated across both randomised and non-randomised populations following novel organ preservation therapy with short-course radiotherapy and transanal endoscopic microsurgery. Although the use of routine transanal endoscopic microsurgery might now be considered over-treatment, at the time of the design of TREC, there were little data to support the long-term oncological safety of a watch-and-wait strategy, and there were concerns that short-course radiotherapy and treatment delay without transanal endoscopic microsurgery represented significant under-treatment.

We conclude that TREC provides evidence to support further evaluation of organ preservation strategies that incorporate short-course radiotherapy as an alternative to radical surgery for early-stage rectal cancer. This is a feasible and acceptable approach for patients. Further evaluation of short-course radiotherapy is justified because of the excellent compliance, low toxicity, benefits in HRQOL, and high organ preservation rates observed in TREC. It is important that organ preservation continues to be evaluated in the context of well-conducted clinical research to establish the oncological safety of this approach.^16^

## Data sharing

Data sharing requests will be considered by the trial management group on written request to trec@trials.bham.ac.uk. De-identified participant data or other pre-specified data will be available, subject to a written proposal and an agreed data sharing agreement.
