# Supplementary material for: Radical surgery versus organ preservation via short-course radiotherapy followed by transanal endoscopic microsurgery for early-stage rectal cancer (TREC): a randomised, open-label feasibility study
Source: Lancet Gastroenterol Hepatol. 2020 Dec 11;6(2):92–105. doi: 10.1016/S2468-1253(20)30333-2 (PMC7802515; doi:10.1016/S2468-1253(20)30333-2)
Supplement: Supplementary appendix [file mmc1.pdf]

THE LANCET
Gastroenterology & Hepatology

Supplementary appendix

This appendix formed part of the original submission and has been peer reviewed. We post it as supplied by the authors.

Supplement to: Bach SP, Gilbert A, Brock K, et al. Radical surgery versus organ preservation via short-course radiotherapy followed by transanal endoscopic microsurgery for early-stage rectal cancer (TREC): a randomised, open-label feasibility study. *Lancet Gastroenterol Hepatol* 2020; published online Dec 10. https://doi.org/10.1016/S2468-1253(20)30333-2.

TREC supplementary tables and figures

Toxicity type	Toxicity grade	Randomised		Non-randomised	
		Organ saving (n=27)	TME surgery (n=28)	Organ saving (n=61)	TME surgery (n=7)
**All toxicity**					
	Grade 1-3	20/27 (80%)	2/3 (67%)	39/60 (75%)	-
	Grade 3	4/27 (15%)	0/3 (0%)	4/60 (7%)	-
**Specific toxicities**					
Anorectal pain	Grade 1-2	1	0	2	-
Constipation	Grade 1-2	1	0	4	-
Cystitis	Grade 1-2	6	0	11	-
Diarrhoea	Grade 1-2	11	2	21	-
	Grade 3	4	0	3	-
Nausea	Grade 1-2	5	0	7	-
Proctitis	Grade 1-2	1	0	3	-
	Grade 3	2	0	2	-
Rectal bleeding	Grade 1	1	0	5	-
Skin reaction	Grade 1-2	7	0	10	-
	Grade 3	0	0	1	-
Vomiting	Grade 1	2	0	3	-

Supplementary table 1: Radiotherapy toxicity by intention-to-treat using CTCAE v4.0 (reported at 3-weeks following completion of SCRT)

Treatment	1st surgery	TNM stage 1st surgery	R	High grade	Ly	V	2nd surgery	TNM stage 2nd surgery	Local recurrence	Distant recurrence	Follow up time (yr)	DFS time (yr)	OS time (yr)
OP	TEM	ypT3Nx	R0	No	No	No	Declined early conversion LAR	n/a	Rectal wall (No salvage)	Para-aortic nodes	5.03	2.83	5.03
OP	TEM	ypT2Nx	R1	No	No	No	Salvaged by low Hartman [declined early conversion]	ypT0N2 (4/23)	Rectosigmoid lymph nodes (resected)	No	5.36	4.49	-
OP	TEM	ypT3Nx	R0	No	No	+	Converted APE	No residual tumour	No	Liver, lung	4.64	4.28	-
OP	TEM	ypT2Nx	R0	Yes	No	+	Converted LAR	ypT2LyV+N0R0	No	Liver & lung	4.96	2.46	-
OP	TEM	ypT3Nx	R0	No	No	No	Declined early conversion APE	n/a	Rectal wall (unfit for salvage)	No	2.57	1.75	-
OP	TEM	ypT2Nx	R0	Yes	No	+	Converted LAR	ypT2LyV+N0R1 (distal margin)	No	Liver, lung & adrenal	2.24	1.19	2.24
TME	LAR	pT2N0	R0	Yes	No	No	n/a	n/a	No	Lung	4.96	2.64	-
TME*	TEM	ypT2Nx	R0	No	No	No	Converted APE	No residual tumour	No	Liver & lung (resected)	4.08	0.35	-

Supplementary table 2: Characteristics of cancer recurrences in the randomised patient population. * randomised to TME but primary treatment was OP (protocol deviation). [Key: TNM – tumour, node, metastasis; R – resection margin; Ly – lymphatic invasion; V – vascular invasion; sm – submucosal tumour stage; APE – abdominoperineal excision]

Treatment	1st surgery	TNM stage 1st surgery	R	High grade	Ly	V	2nd surgery	Local recurrence	Distant recurrence	Follow up time (yr)	DFS time (yr)	OS time (yr)
OP	TEM	ypT2Nx	R0	No	No	+	No	No	Lung	5.85	1.36	-
OP	TEM	ypT1sm1Nx	R0	No	No	No	No	Mesorectal nodes	Liver	1.39	1.30	1.39
OP	TEM	ypT1sm1Nx	R0	No	No	No	Salvaged by APE	Rectal wall at TEM site	No	5.70	4.83	-
OP	TEM	ypT1sm3Nx	R0	No	No	No	No	Rectal wall at TEM site	Liver & lung	3.38	1.25	-
OP	TEM	ypT3Nx	R0	No	No	No	No	No	Liver	3.55	0.74	-
OP	TEM	ypT2Nx	R0	Yes	No	No	Unfit surgical salvage	Mesorectal and extra-mesorectal lymph nodes	No	1.66	1.66	-
OP	TEM	ypT0Nx	R0	No	No	No	Unfit surgical salvage	Rectal wall at TEM site	CT scan not done	2.52	1.41	-
OP	TEM	ypT0Nx	R0	No	No	No	No	No	Liver & lung	1.17	0.99	1.17
OP	TEM	ypT1sm2	R0	Yes	No	No	Salvage APE	Rectal wall and mesorectal nodes: ypT3N1R0	No	2.32	2.11	-

Supplementary table 3: Characteristics of cancer recurrences in the non-randomised patient population
[Key: TNM – tumour, node, metastasis; R – resection margin; Ly – lymphatic invasion; V – vascular invasion; sm – submucosal tumour stage; APE – abdominoperineal excision]

	Baseline		3 months		6 months		12 months		24 months		36 months	
	OP	TME	OP	TME	OP	TME	OP	TME	OP	TME	OP	TME
**EORTC QLQ C30 FUNCTION: higher scores = better function (0-100)**												
Global health status§	83.7 (2.7; 26)	79.5 (4.8; 24)	75.5 (3.5; 18)	63.2 (4.8; 24)	80.4 (3.4; 20)	73.2 (4.1; 23)	78.3 (3.5; 20)	69.9 (4.4; 23)	76.2 (5.3; 20)	69.4 (4.6; 21)	84.5 (1.7; 14)	67.6 (5.9; 18)
Physical functioning§	88.6 (3.3; 26)	85.6 (4.5; 24)	86.0 (3.4; 19)	73.1 (5.7; 24)	85.7 (4.2; 20)	81.2 (4.2; 23)	83.9 (4.4; 20)	78.2 (5.4; 22)	80.0 (5.1; 20)	79.2 (4.4; 21)	88.7 (1.9; 13)	79.0 (5.0; 17)
Role functioning§	93.6 (2.9; 26)	83.3 (6.6; 24)	77.2 (6.4; 19)	60.4 (6.9; 24)	84.2 (6.2; 20)	71.7 (6.1; 23)	87.5 (4.2; 20)	77.3 (6.0; 22)	78.3 (6.4; 20)	76.2 (5.8; 21)	89.7 (4.0; 13)	69.4 (7.2; 18)
Emotional functioning§	85.6 (2.5; 26)	74.0 (5.8; 24)	90.3 (3.4; 18)	74.7 (5.0; 24)	90.0 (2.6; 20)	81.9 (4.8; 23)	88.8 (2.7; 20)	79.5 (5.0; 22)	85.0 (4.2; 20)	81.1 (4.5; 21)	90.5 (2.9; 14)	76.9 (6.1; 18)
Cognitive functioning§	89.7 (3.3; 26)	81.2 (5.1; 24)	89.8 (4.7; 18)	79.2 (6.1; 24)	91.7 (3.3; 20)	85.5 (4.2; 23)	90.4 (2.6; 19)	84.8 (3.6; 23)	87.5 (4.5; 20)	86.7 (4.8; 20)	95.2 (3.7; 14)	76.9 (5.7; 18)
Social functioning§	94.9 (2.4; 26)	86.1 (6.1; 24)	82.4 (5.9; 18)	60.4 (6.9; 24)	90.8 (3.9; 20)	68.1 (6.2; 23)	91.7 (2.6; 20)	71.2 (7.0; 22)	83.3 (5.9; 20)	64.2 (7.8; 20)	90.5 (4.2; 14)	62.0 (6.0; 18)
**EORTC QLQ C30 SYMPTOMS: higher scores = worse symptoms (0-100)**												
Fatigue	16.2 (4.1; 26)	25.0 (5.8; 24)	17.8 (4.7; 19)	36.6 (5.4; 24)	18.9 (5.1; 20)	27.5 (5.0; 23)	15.6 (3.7; 20)	31.3 (5.6; 22)	21.1 (5.7; 20)	30.2 (5.2; 21)	12.0 (3.9; 13)	27.8 (5.6; 18)
Nausea and vomiting	3.2 (2.6; 26)	5.6 (2.2; 24)	1.8 (1.8; 19)	10.4 (4.5; 24)	2.5 (1.8; 20)	5.1 (3.7; 23)	5.8 (3.5; 20)	7.6 (4.2; 22)	5.0 (3.0; 20)	3.3 (1.9; 20)	1.3 (1.3; 13)	13.0 (6.1; 18)
Pain	13.5 (3.9; 26)	16.7 (5.4; 24)	9.6 (4.1; 19)	26.4 (6.6; 24)	16.7 (5.8; 20)	18.1 (5.8; 23)	10.5 (4.5; 19)	19.6 (5.0; 23)	15.0 (3.6; 20)	15.9 (3.7; 21)	9.5 (3.4; 14)	25.0 (5.9; 18)
Dyspnoea	9.0 (3.9; 26)	9.7 (5.1; 24)	7.0 (4.1; 19)	16.7 (5.7; 24)	18.3 (7.0; 20)	11.6 (4.0; 23)	18.3 (5.1; 20)	16.7 (6.1; 22)	13.3 (6.1; 20)	19.0 (7.1; 21)	7.7 (4.1; 13)	16.7 (5.6; 18)
Insomnia	19.2 (4.2; 26)	29.2 (6.7; 24)	24.6 (8.0; 19)	33.3 (6.0; 24)	23.3 (4.9; 20)	27.5 (5.4; 23)	23.3 (5.5; 20)	30.3 (6.2; 22)	20.0 (6.6; 20)	29.8 (7.2; 19)	10.3 (5.8; 13)	37.0 (7.1; 18)
Appetite loss	7.7 (4.6; 26)	15.3 (6.6; 24)	5.3 (2.9; 19)	20.8 (6.0; 24)	6.7 (3.9; 20)	10.1 (4.9; 23)	5.0 (5.0; 20)	13.6 (5.2; 22)	11.7 (4.4; 20)	12.7 (4.3; 21)	2.6 (2.6; 13)	16.7 (6.7; 18)
Constipation	0.0 (0.0; 26)	16.7 (5.3; 24)	5.3 (5.3; 19)	10.1 (4.4; 23)	3.3 (2.3; 20)	14.5 (5.9; 23)	3.3 (2.3; 20)	16.7 (5.3; 22)	10.0 (4.3; 20)	17.5 (4.9; 21)	7.7 (5.5; 13)	14.8 (4.0; 18)
Diarrhoea	11.5 (3.7; 26)	18.1 (5.7; 24)	9.3 (3.6; 18)	16.7 (5.3; 22)	11.7 (4.4; 20)	23.2 (6.8; 23)	8.8 (5.0; 19)	22.2 (7.4; 21)	8.3 (3.3; 20)	17.5 (5.5; 21)	4.8 (3.2; 14)	31.5 (8.3; 18)
Financial problems	1.3 (1.3; 26)	13.9 (6.3; 24)	1.9 (1.9; 18)	22.2 (7.7; 24)	6.7 (5.2; 20)	8.7 (4.3; 23)	1.7 (1.7; 20)	15.9 (5.5; 23)	11.7 (6.1; 20)	12.3 (5.2; 19)	0.0 (0.0; 14)	22.2 (8.1; 18)

Supplementary table 4: EORTC-QLQ C30 PRO data [mean (SD; N)] over time for randomised patients; organ preservation [OP] using SCRT and TEM versus total mesorectal excision [TME]. § Function scores: higher scores = better function.

	Baseline		3 months		6 months		12 months		24 months		36 months	
	OP	TME	OP	TME	OP	TME	OP	TME	OP	TME	OP	TME
**EORTC-QLQ C29 FUNCTION: higher scores = better function (0-100)**												
Body Image§	94.7 (2.0; 25)	76.2 (6.3; 24)	93.6 (2.3; 19)	61.3 (5.6; 24)	94.7 (2.0; 20)	67.9 (4.3; 23)	94.2 (2.0; 19)	69.8 (5.5; 23)	93.3 (2.2; 20)	66.1 (7.2; 21)	92.1 (2.7; 14)	66.4 (7.0; 18)
Health anxiety§	74.7 (4.8; 25)	53.3 (5.8; 25)	82.5 (6.4; 19)	51.4 (6.3; 24)	80.0 (4.5; 20)	66.7 (5.1; 23)	71.7 (5.6; 20)	63.8 (6.3; 23)	71.7 (5.6; 20)	57.1 (7.3; 21)	78.6 (4.4; 14)	68.5 (5.0; 18)
Weight worries§	84.6 (4.2; 26)	70.7 (7.0; 25)	91.2 (3.5; 19)	70.8 (6.4; 24)	95.0 (2.7; 20)	75.4 (7.0; 23)	95.0 (3.6; 20)	75.4 (5.6; 23)	86.7 (3.7; 20)	85.7 (4.9; 21)	85.7 (5.8; 14)	79.6 (6.1; 18)
Male libido (sexual interest)§	43.1 (6.9; 17)	30.6 (9.6; 12)	25.6 (6.7; 13)	13.9 (6.4; 12)	35.7 (5.5; 14)	27.8 (9.9; 12)	31.0 (5.5; 14)	25.0 (7.3; 12)	30.8 (8.8; 13)	33.3 (9.9; 10)	21.2 (6.8; 11)	36.4 (7.0; 11)
Female libido (sexual interest)§	13.3 (13.3; 5)	12.5 (6.1; 8)	0.0 (0.0; 5)	6.7 (4.4; 10)	0.0 (0.0; 4)	16.7 (6.3; 8)	0.0 (0.0; 2)	9.5 (6.1; 7)	11.1 (11.1; 3)	23.8 (6.1; 7)	0.0 (0.0; 2)	5.6 (5.6; 6)
**EORTC-QLQ C29 SYMPTOMS: higher scores = worse symptoms (0-100)**												
Urinary frequency	25.0 (4.7; 26)	34.0 (5.3; 25)	25.0 (6.1; 18)	34.8 (6.3; 23)	25.8 (5.7; 20)	37.1 (5.0; 22)	25.8 (5.1; 20)	31.2 (5.6; 23)	30.7 (5.7; 19)	34.2 (4.9; 20)	21.4 (6.2; 14)	34.3 (5.1; 18)
Blood/Mucous in stools	16.7 (4.0; 26)	29.3 (5.9; 25)	8.8 (3.2; 19)	7.2 (3.3; 23)	6.7 (2.8; 20)	3.2 (1.9; 21)	5.0 (1.8; 20)	9.1 (3.9; 22)	5.8 (3.7; 20)	3.2 (2.2; 21)	2.4 (1.6; 14)	7.4 (2.8; 18)
Stool frequency	9.4 (2.5; 23)	13.5 (3.9; 21)	26.5 (5.2; 17)	18.8 (4.1; 23)	18.4 (4.4; 19)	39.4 (6.7; 22)	18.5 (4.0; 18)	31.1 (4.9; 22)	14.6 (3.7; 16)	26.3 (5.5; 19)	14.3 (4.2; 14)	30.6 (5.6; 18)
Urinary incontinence	3.8 (2.8; 26)	10.7 (5.0; 25)	5.6 (3.0; 18)	17.4 (5.9; 23)	5.0 (2.7; 20)	16.7 (5.7; 22)	3.5 (2.4; 19)	23.2 (6.1; 23)	14.0 (5.3; 19)	15.0 (4.5; 20)	2.4 (2.4; 14)	14.8 (4.0; 18)
Dysuria	1.3 (1.3; 26)	4.2 (4.2; 24)	0.0 (0.0; 18)	13.0 (5.4; 23)	3.3 (2.3; 20)	6.3 (2.9; 21)	3.3 (2.3; 20)	8.7 (3.8; 23)	8.3 (4.1; 20)	1.7 (1.7; 20)	2.4 (2.4; 14)	1.9 (1.9; 18)
Abdominal pain	5.1 (3.6; 26)	13.9 (4.4; 24)	3.5 (2.4; 19)	18.8 (5.1; 23)	3.3 (2.3; 20)	9.1 (3.9; 22)	5.0 (2.7; 20)	14.5 (4.1; 23)	6.7 (3.1; 20)	15.9 (4.4; 21)	4.8 (3.2; 14)	16.7 (5.6; 18)
Buttock pain	3.8 (2.1; 26)	13.9 (5.3; 24)	14.0 (5.9; 19)	26.1 (6.3; 23)	10.0 (4.3; 20)	16.7 (5.3; 22)	5.0 (2.7; 20)	20.3 (5.8; 23)	13.3 (4.5; 20)	20.6 (5.4; 21)	7.1 (3.8; 14)	18.5 (6.2; 18)
Bloating	11.5 (3.7; 26)	18.7 (5.8; 25)	8.8 (3.5; 19)	26.4 (6.0; 24)	13.3 (3.7; 20)	16.7 (4.2; 22)	11.7 (4.4; 20)	26.1 (6.6; 23)	6.7 (3.9; 20)	11.7 (4.4; 20)	7.7 (4.1; 13)	22.2 (5.4; 18)
Dry mouth	11.5 (4.5; 26)	21.3 (6.0; 25)	8.8 (4.3; 19)	22.2 (5.6; 24)	13.3 (5.1; 20)	24.6 (7.3; 23)	13.3 (4.5; 20)	23.2 (6.4; 23)	20.0 (6.6; 20)	17.5 (5.5; 21)	14.3 (4.6; 14)	20.4 (7.2; 18)
Hair loss	1.3 (1.3; 25)	1.3 (1.3; 25)	7.0 (3.2; 19)	9.7 (4.2; 24)	3.3 (2.3; 20)	12.1 (5.2; 22)	0.0 (0.0; 20)	3.0 (2.1; 22)	1.7 (1.7; 20)	3.3 (2.3; 20)	4.8 (4.8; 14)	7.4 (5.8; 18)
Taste change	2.6 (1.8; 26)	8.0 (4.8; 25)	0.0 (0.0; 19)	15.3 (5.3; 24)	1.7 (1.7; 20)	7.6 (3.0; 22)	3.3 (2.3; 20)	13.0 (5.0; 23)	6.7 (3.9; 20)	9.5 (4.7; 21)	2.4 (2.4; 14)	3.9 (2.7; 17)
Flatulence	23.2 (4.9; 23)	35.0 (6.6; 20)	29.2 (7.4; 16)	33.3 (4.9; 22)	21.1 (6.4; 19)	42.0 (4.8; 23)	29.6 (6.0; 18)	42.4 (6.6; 22)	31.2 (6.4; 16)	40.0 (5.7; 20)	33.3 (7.8; 14)	40.7 (6.9; 18)
Faecal incontinence	2.9 (2.0; 23)	5.0 (2.7; 20)	17.6 (7.6; 17)	24.6 (5.2; 23)	15.8 (6.4; 19)	30.3 (5.8; 22)	17.6 (7.1; 17)	15.9 (4.4; 21)	12.5 (4.2; 16)	19.3 (6.4; 19)	19.0 (5.8; 14)	25.9 (8.8; 18)
Sore skin	4.3 (3.2; 23)	7.9 (3.2; 21)	19.6 (7.6; 17)	27.5 (5.4; 23)	19.3 (6.9; 19)	34.8 (6.4; 22)	7.4 (3.4; 18)	22.2 (5.3; 21)	8.3 (4.8; 16)	19.3 (6.4; 19)	7.1 (7.1; 14)	24.1 (5.3; 18)
Embarrassment about bowel function	5.8 (2.7; 23)	11.7 (5.6; 20)	17.6 (8.1; 17)	34.8 (6.8; 23)	12.3 (5.8; 19)	36.2 (7.2; 23)	16.7 (6.2; 18)	31.7 (7.1; 21)	10.4 (5.9; 16)	33.3 (7.3; 20)	7.1 (3.8; 14)	38.9 (7.3; 18)
Impotence	31.4 (7.8; 17)	21.2 (10.3; 11)	36.4 (11.4; 11)	25.0 (11.7; 12)	42.9 (8.1; 14)	55.6 (13.8; 12)	51.3 (10.4; 13)	48.5 (12.2; 11)	48.1 (14.8; 9)	51.9 (16.8; 9)	48.5 (13.8; 11)	53.3 (14.2; 10)
Dyspareunia	0.0 (0.0; 4)	0.0 (0.0; 5)	8.3 (8.3; 4)	11.1 (11.1; 3)	0.0 (0.0; 2)	16.7 (16.7; 2)	0.0 (0.0; 2)	11.1 (11.1; 3)	22.2 (22.2; 3)	11.1 (11.1; 3)	0.0 (0.0; 2)	13.3 (8.2; 5)

Supplementary table 5: EORTC-QLQ C29 PRO data [mean (SD; N)] over time for randomised patients; organ preservation [OP] using SCRT and TEM versus total mesorectal excision [TME]. § Function scores: higher scores = better function.

EORTC QLQ C30	Baseline	3 months	6 months	12 months	24 months	36 months
FUNCTION ITEMS	Percentage probability of benefit of OP vs TME surgery					
Global health status§	73.5	98.6	93.8	95.8	94.6	94
Physical functioning§	81.7	93	75.1	89.5	85.7	81.7
Role functioning§	82.7	98.8	95.7	95	69	95
Emotional functioning§	98.4	99.7	97.3	99.3	95.9	97.3
Cognitive functioning§	95.8	95.3	85.8	92.3	82.3	98.2
Social functioning§	92	99.7	99.8	100	99.4	99.7
SYMPTOMS ITEMS						
Fatigue	93.7	99.5	88.3	99.5	87.1	89
Nausea and vomiting	74	96.6	75	74.3	41.4	97.7
Pain	73.3	97.6	51.5	88.6	52.1	80.9
Dyspnoea	63.1	88.6	21.5	54.2	77	72.8
Insomnia	94.9	82.8	75.6	87.4	92	97
Appetite loss	90.2	98.8	80.3	97.5	58.2	93.3
Constipation	99.8	78.6	96.7	99.6	88.7	81.8
Diarrhoea	89.6	85.1	93.6	97.8	92.3	99.4
Financial problems	98.6	99.8	66.9	99.1	75.8	99

Supplementary table 6: Bayesian model analysis of EORTC QLQ C30 for randomised patients. § Function scores. FUNCTION: Probability that the mean score under SCPRT + TEMS is greater than that under radical surgery (adjusted for assessment timepoint and patient baseline value). SYMPTOMS: Probability that the mean score under SCPRT + TEMS is less than that under radical surgery (adjusted for assessment timepoint and patient baseline value). Note analysis not possible for female libido or dyspareunia items.

EORTC QLQ C29	Baseline	3 months	6 months	12 months	24 months	36 months
**FUNCTION ITEMS**	**Percentage probability of benefit of OP vs TME surgery**					
Body Image§	99.7	100	100	100	100	99.6
Health anxiety§	99.9	100	98	94.2	97.7	92.9
Weight worries§	94.8	96.9	99.7	100	58.3	69.5
Male libido (sexual interest)§	92.5	98	71.2	82	56.4	22.3
**SYMPTOM ITEMS**						
Urinary frequency	93.5	76.9	72	56.2	48.3	68.1
Blood/Mucous in stools	96.1	11.7	4.5	49.5	15.7	78.8
Stool frequency	89.7	27.7	99.9	98.9	98.2	96.6
Urinary incontinence	96.5	94.5	94.5	100	64.8	96.4
Dysuria	67.9	100	79.2	89.4	8.4	40.8
Abdominal pain	99.4	99.9	92.2	96.5	96.7	93.3
Buttock pain	97.2	93.5	80.2	98.5	83.8	68.3
Bloating	88.5	98.9	76.7	98.1	79.3	93.5
Dry mouth	92.2	97.9	90.2	96.5	42.7	47.7
Hair loss	36.1	43.9	84.7	99.1	55.2	31.9
Taste change	90.2	100	95.5	97.9	80.3	67
Flatulence	97.4	73.7	99.1	92.3	89	51.6
Faecal incontinence	93.3	81.8	96.2	67	83.6	51.5
Sore skin	95.3	91	97.4	99.7	96.6	99.9
Embarrassment about bowel function	98.8	98	99.4	98.9	99.8	99.9
Impotence	5	13.4	47.1	24	48.3	55.7

Supplementary table 7: Bayesian model analysis of EORTC QLQ CR29 for randomised patients. § Function scores. FUNCTION: Probability that the mean score under SCPRT + TEMS is greater than that under radical surgery (adjusted for assessment timepoint and patient baseline value). SYMPTOMS: Probability that the mean score under SCPRT + TEMS is less than that under radical surgery (adjusted for assessment timepoint and patient baseline value). Note analysis not possible for female libido or dyspareunia items.

Supplementary figure 1: cumulative recruitment of randomised and non-randomised (registered) patients according to site.

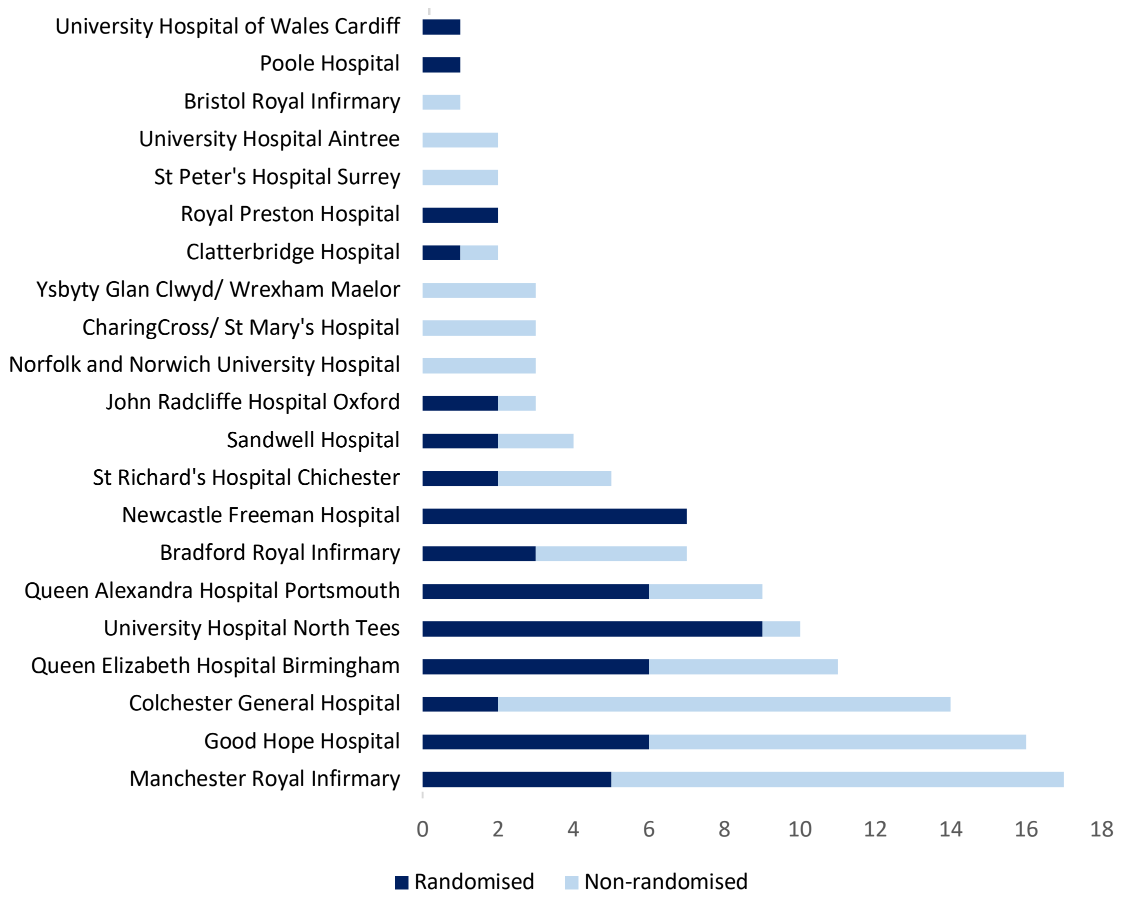

Supplementary figure 2: CONSORT diagram of study patients and complete PRO forms. MDT: Multidisciplinary team; SCPRT: Short course pre-operative radiotherapy; TEMS: Transanal endoscopic microsurgery; TME: Total mesorectal excision

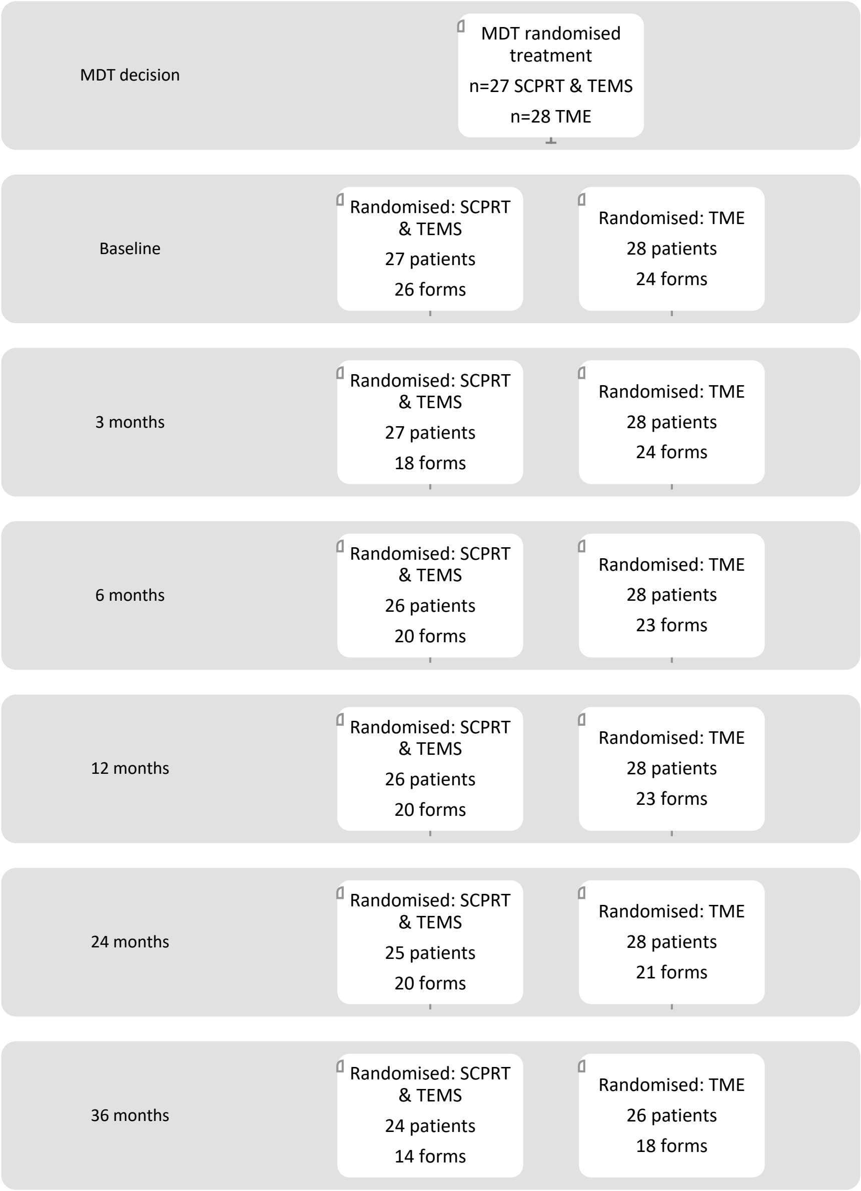

TREC Collaborative Group

Chief Investigator: Simon Bach (University Hospital Birmingham NHS Foundation Trust)

Trial management committee:
Simon Bach, Gina Brown, Christopher Cunningham, Ian Geh, Richard Gray, Kelly Handley, Jim Hill, Stephan Korsgen, Pauline Leonard, Laura Magill, John R T Monson, Neil J Mortensen, Dion G Morton, Arthur Sun-Myint, Phil Quirke, David Sebag-Montefiore, Nick West, Elizabeth Southgate.

Independent patient advocate: Ann Russell

University of Birmingham Clinical Trials Unit: Peter Antonio, Manjinder Kaur, Laura Magill, Alex Vince, (trial management); Nick Hilken, Chakanaka Sidile, Adrian Wilcockson (programming); Kelly Handley, Natalie Marchevsky (statistics).

Data Monitoring and Ethics Committee: Richard Peto (Chair), Tom Crosby, Brendan Moran, Julie Olliff.

Participating centres and investigators (*principal investigator at each centre): Aintree University Hospitals NHS Foundation Trust; Katti Ashok, Simone Slawik*, Andrew Smethurst, Rajaram Sripadam, Veena Tagore, Monica Terlizzo, Ashford and St Peter's Hospitals NHS Foundation Trust; Bearn Philip E, Robert Davies, Susan Dodd, Sharadah Essapen, Pasha Nisar*, Alexandra Stewart, Jonathan Trickett, Besti Cadwaladr University Health Board; Bansal Ashish, Peter J Billings, Palanichamy Chandran, Conor Corr, Edward Favill, Simon Gollins*, Peter Marsh, Andrew Maw*, Rekha Neupane, Ramesh Rajagopal, Bradford Teaching Hospitals NHS Foundation Trust; Cooper Rachel A, John Philip Griffith, Paul Hatfield, Andy Lowe, Julian Ostrowski, Jonathan Robinson, Rhian Simpson, Mark Steward*, Cardiff and Vale University Health Board; Adams Richard, Robert E Bleehen, Michael Davies*, Meleri Morgan, East Suffolk and North Essex NHS Foundation Trust; Boone Darren, Nicola Lacey, Ian Seddon, Bruce Sizer, Helen Stunell, Matthew Tutton*, Shaobin Wu, Frimley Health NHS Foundation Trust; Hadaki Maher, Imperial College Healthcare NHS Trust; Blunt Dominic, Susan Cleator, Ara Darzi, Robert Goldin, Paul Ziprin*, Lancashire Teaching Hospitals NHS Foundation Trust; Alan Beveridge* Mike Dobson, Mark A Pitt, Shabbir Susnerwala, Deborah Williamson, Manchester University NHS Foundation Trust; Jim Hill* Georgina Howarth, Stephen Lee, Paul Wright, Newcastle Upon Tyne Hospitals NHS Foundation Trust; Paul J Hainsworth* Tim Hoare, Alan F Horgan, Fiona McDonald, Stephanie J Needham, John Scott, Timothy Simmons, Norfolk & Norwich University Hospitals NHS Foundation Trust; Biswas Debashis, James Hernon*, Gaurav Kapur, Sandeep Kapur, James Sington, Christopher Speakman, William Stebbings, Stuart Williams, North Tees and Hartlepool NHS Foundation Trust; Adusumalli Madhavi, Anil K Agarwal, David Borowski,

Dharmendra Kumar Garg, Talvinder Gill*, Mohammed Hegab, Catherine L Hobday, Veena Rao, Jyotsna J Shrimankar, Mohamed Amin Tabaqchali, David Wilson, Oxford University Hospitals NHS Foundation Trust; Christopher Cunningham* Oliver Jones, Neil J Mortensen, Andrew Slater, Aron Szuts, Lai Mun Wang, Bryan F Warren, Andrew Weaver, Poole Hospital NHS Foundation Trust; Ahmad Mukhtar, Julian Alexander, Maxine Flubacher*, David Tarver, Portsmouth Hospitals University NHS Trust; Baluch Suhail, Richard Beable, David Cowlishaw, Antony Higginson, Jim Khan*, Prokopios Vogiatzis, Sandwell and West Birmingham NHS Trust; Neil Cruickshank* Howard Joy, David Peake, Ulises Zanetto, The Christie NHS Foundation Trust; Mark Saunders* The Clatterbridge Cancer Centre NHS Foundation Trust; Arthur Sun Myint*, Rajaram Sripadam, The Leeds Teaching Hospitals NHS Trust; Cooper Rachel, Paul Hatfield, Phil Quirke, David Sebag-Montefiore*, Mark Teo, Nick West, University Hospitals Birmingham NHS Foundation Trust; Allan Arthur, Simon Bach*, Ian Geh, John Glaholm, Mark Goldstein, Rahul Hejmadi, Stephan Korsgen*, Gerald Langman, Dion G Morton, Cyril A Nelson, Deborah Tattersall, University Hospitals Bristol NHS Foundation Trust; Falk Stephen, Robert Longman, Huw Roach, Jamshed Shabbir, Golda Shelley-Fraser , Michael G Thomas*, Western Sussex Hospitals NHS Foundation Trust; Cripps Neil, Yasser Haba, Guy Harris, Max Hookway, Bruce Levy, Jay N L Simson*, Angela Skull, Tijjani Umar.
